# Thromboxane A_2_ Receptor Stimulation Enhances Microglial Interleukin-1β and NO Biosynthesis Mediated by the Activation of ERK Pathway

**DOI:** 10.3389/fnagi.2016.00008

**Published:** 2016-01-29

**Authors:** Wanlin Yang, Aijuan Yan, Tingting Zhang, Jiaxiang Shao, Tengyuan Liu, Xiao Yang, Weiliang Xia, Yi Fu

**Affiliations:** ^1^Department of Neurology and Institute of Neurology, Rui Jin Hospital, School of Medicine, Shanghai Jiao Tong UniversityShanghai, China; ^2^School of Biomedical Engineering and Med-X Research Institute, Shanghai Jiao Tong UniversityShanghai, China

**Keywords:** thromboxane A_2_ receptor, cerebral ischemia, microglia, inflammation, ERK

## Abstract

**Background and Purpose:** Thromboxane A_2_ (TXA_2_) receptors (TP) interact with the ligand TXA_2_ to induce platelet aggregation and regulate hemostasis. Recently TP-mediated signaling has been suggested to function in multiple cell types in the brain. In this report, we aim to study the expression and physiological role of TP in microglia, in particular after brain ischemia.

**Methods:** Ischemic brain sections were analyzed for TP expression. Microglial cell line and primary microglia were cultured, or neuronal cell line co-culture system was used to determine the TP mediated signaling in inflammation and microglia activation.

**Results:** We found that the TP level was significantly increased in ipsilateral mouse brain tissue at 24 h after ischemia-reperfusion, which was also found to partly co-localize with CD11b, a marker for microglial and infiltrated monocyte/macrophage, in peri-infarct area. Immunofluorescence staining of primary microglia and microglial cell line BV2 revealed the predominant membrane distribution of TP. Conditioned culture media from TP agonist U46619-treated BV2 cells decreased neuronal SH-SY5Y cell viability and induced apoptotic morphological changes. Furthermore, U46619 enhanced IL-1β, IL-6, and iNOS mRNA expression as well as IL-1β and NO releases in BV2 cells or primary microglia. Such stimulation could be attenuated by TP antagonist SQ29548 or MEK inhibitor U0126. The dose- and time-dependent extracellular-signal-regulated kinase (ERK) phosphorylation induced by U46619 further demonstrated ERK signaling-mediated microglia activation by TP agonist.

**Conclusion:** This study has shown a novel role of TP in microglia activation via the ERK signaling pathway, which provides insights for the management of neuroinflammation in diseases like cerebral infarction.

## Introduction

Cerebral infarction (CI), which accounts for 88% of all strokes, is an irreversible brain injury resulting from a disturbance in blood supply to cerebral tissue, characterized by high morbidity, disability, and mortality rate (Moskowitz et al., 2010). There is increasing evidence that post-ischemic inflammation plays an important role in ischemia brain injury (Ceulemans et al., 2010). Microglia are the resident macrophages in central nervous system, which plays a critical role in the post-ischemic inflammation of brain ischemia (Kawabori and Yenari, 2014). Overly activated microglia will release pro-inflammatory cytokines, chemokines, reactive oxygen species, and nitric oxide (NO), leading to more severe brain damage (Lai and Todd, 2006).

Thromboxane A_2_ (TXA_2_) is an arachidonic acid metabolite synthesized by cyclooxygenase and thromboxane synthase (Hamberg et al., 1975). Its chemical half-life is ∼30 s and it is rapidly degraded into an inactive form of thromboxane B_2_ (TXB_2_). Therefore, TXA_2_ regulates the functions of cells around the TXA_2_-producing cells by an autocrine or paracrine manner (Nakahata, 2008). Experimental and clinical studies have demonstrated that there is a local increase of TXA_2_ biosynthesis in CI (Nusing and Ullrich, 1990; Fisher, 1993). Activated microglia are the principal source of brain-derived TXA_2_ (Giulian et al., 1996). TXA_2_ exerts its action by binding to the specific G protein-coupled TP (Hirata et al., 1991). In the brain, many types of cells express the TP, such as microglia (Kitanaka et al., 1995), astrocytes (Nakahata et al., 1995), and oligodendrocytes (Blackman et al., 1998). The distribution of the TP in the brain indicates that it may be involved in a wide range of central nervous system diseases. Previous studies have shown that gene polymorphism of the TP is associated with CI (Kaneko et al., 2006; Zhao et al., 2013; Wang et al., 2014). Under pathological conditions, such as subarachnoid hemorrhage, the TP level is up-regulated in the brain (Ansar et al., 2010). Furthermore, stimulation of the TP in astrocytes resulted in secretion of interleukin 6 (IL-6; Obara et al., 2005). In oligodendrocytes, the TP was involved in enhanced proliferation and survival (Blackman et al., 1998; Lin et al., 2005; Ramamurthy et al., 2006). In Schwann cells, TP activation promotes cyclic adenosine monophosphate (cAMP) and cAMP-response-element-binding protein (CREB) phosphorylation (Muja et al., 2001). However, the physiological function of TXA_2_ signaling in microglia has not been investigated.

The objective of this study is to examine the role of TP in the regulation of microglia inflammation and related mechanism, thus providing new insights for the prevention and treatment of CI.

## Materials and Methods

### Reagents and Animals

Thromboxane A_2_ receptors agonist U46619 and antagonist SQ29548 were purchased from Sigma Aldrich (St. Louis, MO, USA). MEK inhibitor U0126 was from Cell Signaling (Beverly, MA, USA). Anti-phospho-ERK antibody and anti-ERK1/2 antibody were from Cell Signaling (Beverly, MA, USA). Anti-CD11b antibody was from BD Biosciences (San Jose, CA, USA). TP antibody was from Santa Cruz Biotechnology (Santa Cruz, CA, USA). Iba1 antibody was from BD Biosciences (San Jose, CA, USA). The Cell Counting kit-8 (CCK-8) was from KeyGen Biotech Co., Ltd. (Nanjing, China). NO assay kit was from Beyotime Institute of Biotechnology (Haimen, China). Adult male ICR mice and 24 h neonatal Sprague–Dawley (SD) rats were purchased from Shanghai SLAC Laboratory Animal Corporation (Shanghai, China). All animal procedures were carried out strictly within national guidelines and approved by the Institutional Animal Care and Use Committee of Shanghai Jiao Tong University.

### Cell Culture

The microglia cell line BV2 and human neuroblastoma cell line SH-SY5Y were provided by Institute of Neurology, Rui Jin Hospital (Shanghai, China). BV2 cells were cultured in Dulbecco’s modified Eagle’s medium (DMEM) supplemented with 10% heat-inactivated (56°C, 30 min) fetal bovine serum (GIBCO), 1% penicillin/streptomycin (GIBCO). SH-SY5Y cells were grown in DMEM supplemented with 10% fetal bovine serum, 1% penicillin/streptomycin. Cells were maintained in a 5% CO_2_ humidified incubator at 37°C. Primary microglia and astrocytes were prepared from 24 h neonatal SD rat brains and then cultivated in DMEM/F12 supplemented with 10% fetal calf serum, penicillin, and streptomycin, as previously described (Jana et al., 2007; Tamashiro et al., 2012).

### Real-Time PCR

Total RNA from BV2 cells was extracted by using Trizol Reagent (Invitrogen, Carlsbad, CA, USA). Reverse transcription of RNA to cDNA was carried out using a PrimeScript RT reagent kit (TaKaRa). Quantitative real-time PCR was performed with SYBR Premix Ex Taq (TaKaRa). Primers had the following sequences: IL-1β (sense 5′-gcaactgttcctgaactcaact-3′ and anti-sense 5′-atcttttggggcgtcaact-3′); IL-6 (sense 5′-tagtccttcctaccccaatttcc-3′ and anti-sense 5′-ttggtccttagccactccttc-3′); iNOS (sense 5′-atgtccgaagcaaacatcac-3′ and anti-sense 5′-taatgtccaggaagtaggtg-3′); GAPDH (sense 5′-aggtcggtgtgaacggatttg-3′ and anti-sense 5′-tgtagaccatgtagttgaggtca-3′). PCR was carried out as follows: denaturing at 95°C for 10 seconds, the following 40 cycles of 95°C for 5 s and 60°C for 30 s. Data were analyzed by using the comparative threshold cycle (Ct) method, where Ct is the number of PCR cycles performed in one sample at a specific point of time, and the results were expressed as fold difference normalized to GAPDH.

### Enzyme-Linked Immunosorbent Assay (ELISA)

IL-1β ELISA was performed following the protocol of ELISA Kit (eBioscience). In brief, BV2 cells were seeded to 12-wellplates and cultured overnight in DMEM without fetal bovine serum. The cells were cultured for 24 h in the presence of U46619, and then the conditioned media were collected, centrifuged at 3000 rpm for 10 min, and the supernatant was subjected to ELISA. The absorbance at 450 nm was measured by using a microplate reader (Synergy2, BioTek). The IL-1β concentration in the conditioned media was calculated using the standard and normalized against the total protein concentration of the same sample.

### Analysis of Cell Viability

The cell viability was determined by CCK-8 kit according to the manufacture’s instructions (Liu et al., 2013). BV2 cells were plated into 6-well plates and incubated for 24 h in the presence of U46619, and then the cell-free BV2 conditioned media were collected, which was applied to neuronal SH-SY5Y cells that had been seeded in 96-well plates. After 24 h, CCK-8 was used to evaluate the changes in SH-SY5Y cell viability. Subsequently, 2 h prior to measuring the absorbance, 10 μl CCK-8 solution in 90 μl growth medium was added to each well. The optical density was determined at an absorbance of 450 nm using a microplate reader (Synergy2, BioTek). Morphological change of SH-SY5Y cell was examined under the phase-contrast microscopy.

### Western Blotting

Western analysis was performed as described previously (Xie et al., 2013). Cells were lysed in RIPA buffer (Millipore, Temecula, CA, USA) containing Complete Protease Inhibitor Cocktail, Phosphatase Inhibitor Cocktail and 2 mM PMSF. Thirty microgram of total protein was electrophoresed through a 10% SDS-polyacrylamide gel electrophoresis. Proteins were transferred electrically from the gel onto a 0.45 μm nitrocellulose membrane (Millipore, Temecula, CA, USA) by the semidry electro-transfer method. The membranes were blocked for 1 h with 5% skim milk and incubated with primary antibodies at 4°C overnight (anti-phospho-ERK antibody and anti-ERK antibody). The blots were washed several times and then incubated at room temperature for 1 h with HRP-conjugated secondary antibody (1:5000 dilution, Epitomics, China). Finally, protein signals were visualized using an ECL detection system (Thermo Scientific). The densities of the bands were analyzed by densitometry using a Gel-Pro Analyzer (Media Cybernetics, Bethesda, MD, USA).

### Transient Middle Cerebral Artery Occlusion (tMCAO) Model

Adult male ICR mice were randomly assigned to the tMCAO group and sham group. Transient MCAO model in mice was performed as previously described (Yang et al., 1994; Liu et al., 2014). Briefly, adult male ICR mice weighing 25–30 g were anesthetized with ketamine (100 mg/kg) and xylazine (10 mg/kg) intraperitoneally. Then the surgical procedure of tMCAO was performed as follows: 6-O suture (Dermalon, 1741-11, Covidien, Cincinnati, OH, USA) coated with silicone was inserted from the left external carotid artery into the internal carotid artery and reached to the origin of middle cerebral artery. With the help of laser Doppler flowmeter (Moor Instruments, Devon, England), successful occlusion was characterized as a reduction of cerebral blood flow (CBF) down to 10% of baseline. After 1.5 h, the suture was removed for reperfusion. Sham operation group (*n* = 5) underwent the same procedure except suture insertion. All of the animals were sacrificed 24 h after reperfusion.

### Immunohistological Staining

Brain cryosections (20 μm in thickness) were fixed for about 10 min with ice-cold 4% paraformaldehyde and thereafter rehydrated in phosphate buffered-saline (PBS, pH 7.2) for 3 × 5 min. The cryosections were then blocked with 10% normal donkey serum and incubated overnight at 4°C with the following primary antibodies: mouse anti-CD11b antibody (1:100 dilution); rabbit anti-TP antibody (1:150 dilution). Subsequently, cryosections were washed with PBS for 3 × 10 min and incubated with Alexa-488-conjugated or Alexa-594-conjugated secondary antibody (1:200 dilution, Life Technologies) for 1 h at room temperature. After nuclei were stained with 4, 6-diamidino-2-phenylindole (DAPI; 1:500 dilution, Beyotime Institute of Biotechnology, China), the fluorescence images were acquired using a fluorescence microscope (Leica, Germany). BV2 cells and Primary microglia cells were fixed with 4% paraformaldehyde and stained with anti-TP antibody (1:150 dilution) and Iba1 antibody (1:100 dilution) at 4°C overnight, followed by incubation with the fluorescent-conjugated secondary antibody.

### Statistical Analysis

GraphPad Prism6 statistical software was used for statistical analysis in this study. When multiple groups were compared, data from three or more independent experiments were analyzed using one-way ANOVA and Tukey’s post-test for multiple comparison. All data were expressed as mean values ± SEM. *P* < 0.05 was considered statistically significant.

## Results

### Increased Expression of the TP After Ischemia-Reperfusion Injury

**FIGURE 1:**
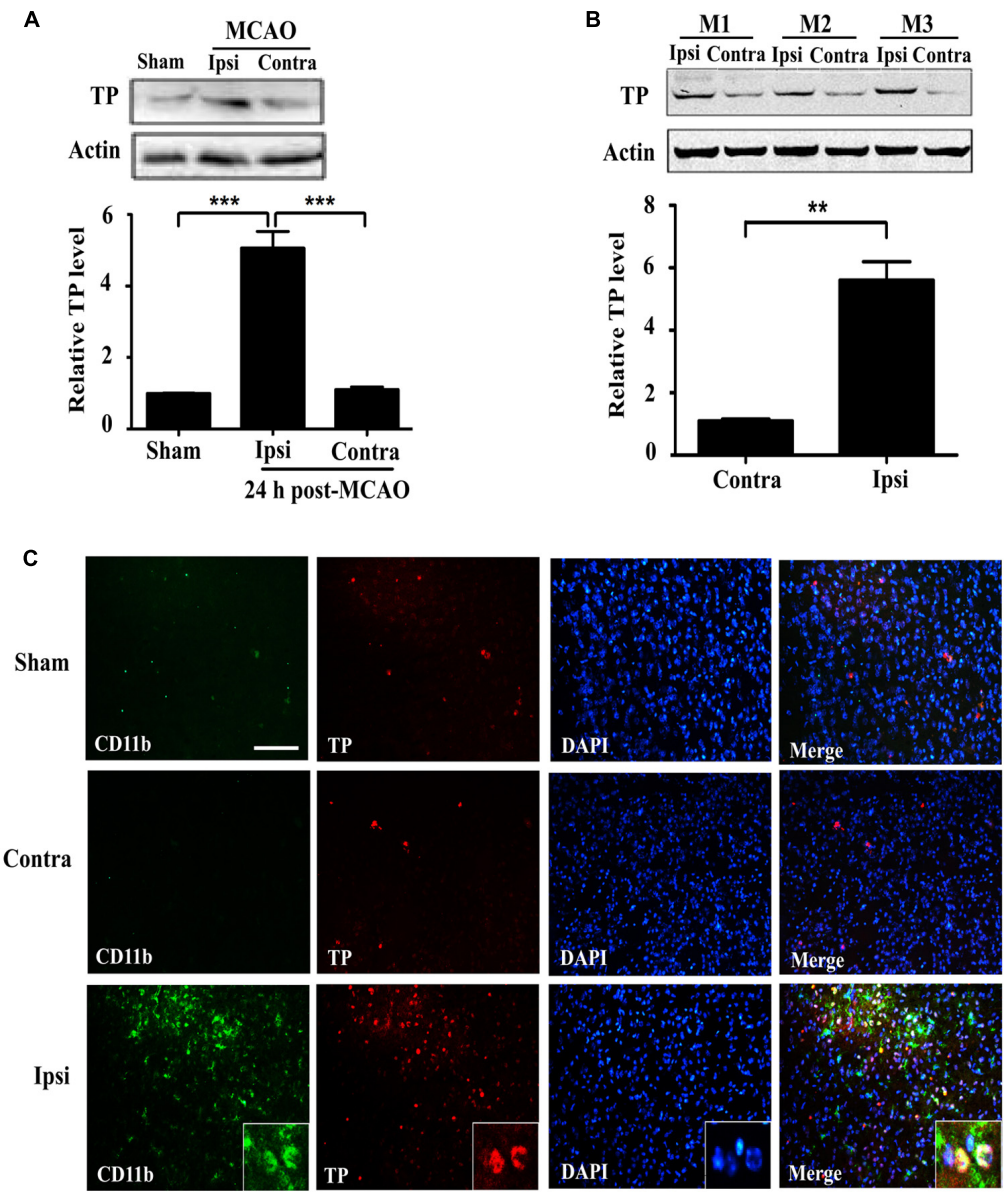
**Expression of the Thromboxane A_2_ receptors (TP) increases in the mouse brain at 24 h after ischemia-reperfusion. (A)** Protein levels of the TP in sham and transient middle cerebral artery occlusion (tMCAO) group at 24 h after ischemia-reperfusion were determined by Western blot, which were quantified in the Bar chart (bottom). Ipsi, damaged hemisphere; Contra, undamaged hemisphere. Five mice per group. Data were normalized against protein level of the sham group. Data were presented as mean ± SEM. **(B)** Protein levels of the TP in ipsilateral and contralateral hemisphere of tMCAO group at 24 h after ischemia-reperfusion from three different animals were analyzed by Western blot and quantified as in the Bar chart (bottom). Data were normalized against protein level of the contralateral hemisphere. Data were presented as mean ± SEM, *n* = 3. **(C)** Immunofluorescence staining of CD11b and TP together with nuclei (DAPI) in the striatum of the ipsilateral and contralateral hemisphere. Bar = 100 μm. Five mice per group. (^∗∗^*P* < 0.01, ^∗∗∗^*P* < 0.001, by one-way ANOVA followed by *post hoc* Tukey’s test).

In order to ascertain whether ischemia-reperfusion enhanced TP expression, we analyzed brain tissues (striatum) from mice that underwent tMCAO. TP protein was significantly increased in the injured ipsilateral brain tissue compared with contralateral hemisphere of the same animal, or with the sham at 24 h after ischemia-reperfusion (**Figures 1A,B**). To further determine the expression of TP in the brain, we performed TP immunofluorescence staining in the mouse brain sections and the result showed that the CD11b signal, a marker for microglial and infiltrated monocyte/macrophage (Akiyama and McGeer, 1990; Mazzone and Ricevuti, 1995), was remarkably up-regulated in the peri-infarct zone of ipsilateral hemisphere at 24 h after ischemia-reperfusion compared with that in contralateral hemisphere or the sham (**Figure 1C**). Furthermore, there was also an up-regulation of TP signals in the penumbra of ipsilateral side that mostly appeared to be co-localized with CD11b, suggesting that at least the activated microglia and infiltrated monocyte/macrophage might contribute significantly to the increase of TP levels after ischemia-reperfusion (**Figure 1C**).

### Microglial TP Localized Primarily on the Cytoplasmic Membrane

**FIGURE 2:**
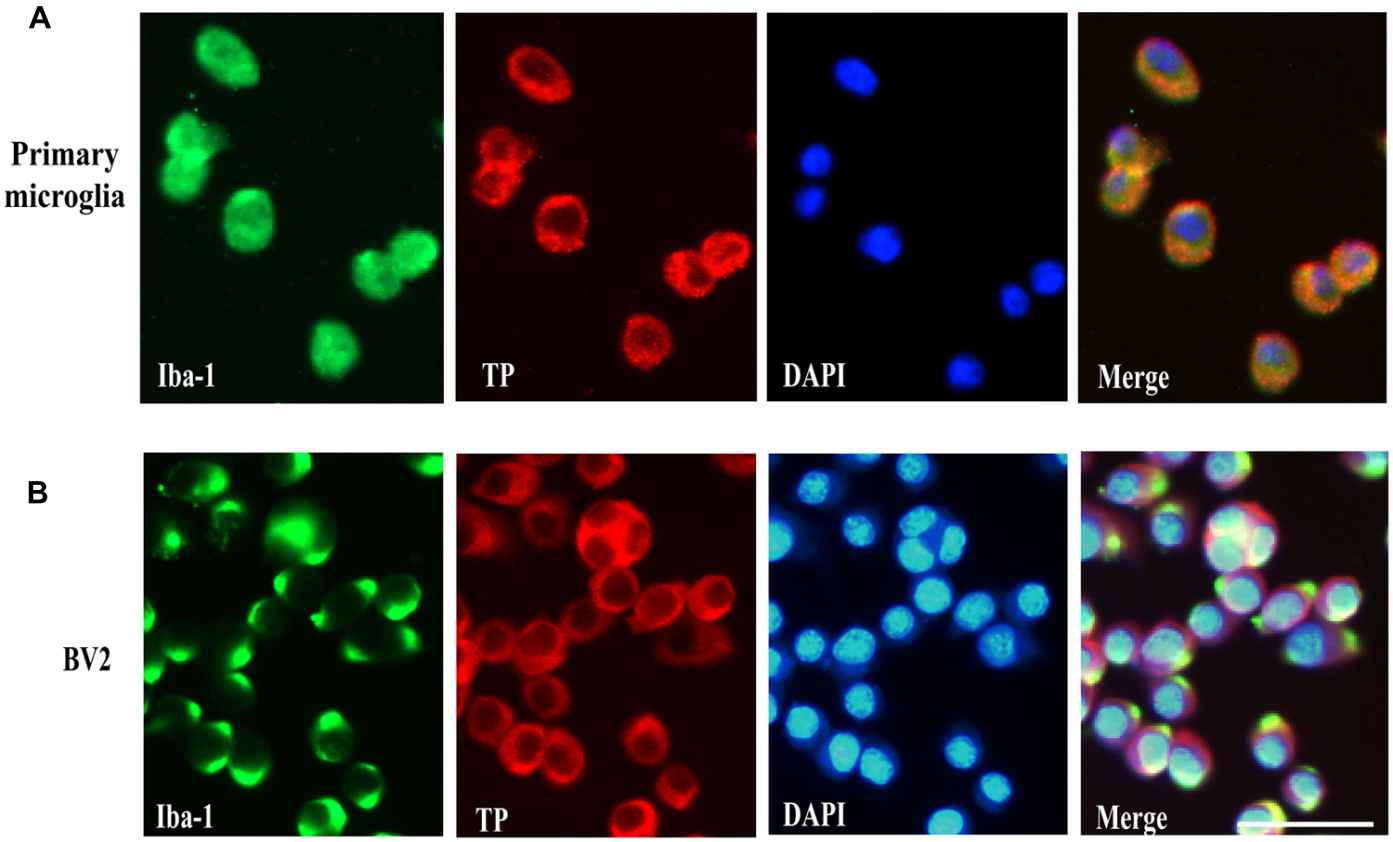
**Cell surface localization of the TP in microglia cells.** Immunofluorescence staining of the TP together with microglia marker Iba-1 in primary microglia cells **(A)** and BV2 cells **(B)**. Bar = 50 μm.

Previous reports have demonstrated TP was localized in the plasma membrane and perinuclear compartments in oligodendrocytes (Ramamurthy et al., 2006). However, the cellular distribution of the TP in microglia cells is presently unknown. To determine whether primary microglia and BV2 cells expressed TP and their distribution, immunofluorescence staining was performed. Primary microglia were stained positively for the microglia marker Iba-1, confirming their identity (**Figure 2A**). The TP was primarily localized on the cytoplasmic membrane, and also in the perinuclear compartments of the cytosol in primary microglia (**Figure 2A**). In BV2 cells, similar staining pattern was observed, where TP was detected predominantly on the cytoplasmic membrane (**Figure 2B**). These results indicated that both primary microglia and BV2 cells expressed TP, which were primarily localized on the cytoplasmic membrane.

### BV2 Microglia After TP Activation Induced Neurotoxicity in Neuronal SH-SY5Y Cells

**FIGURE 3:**
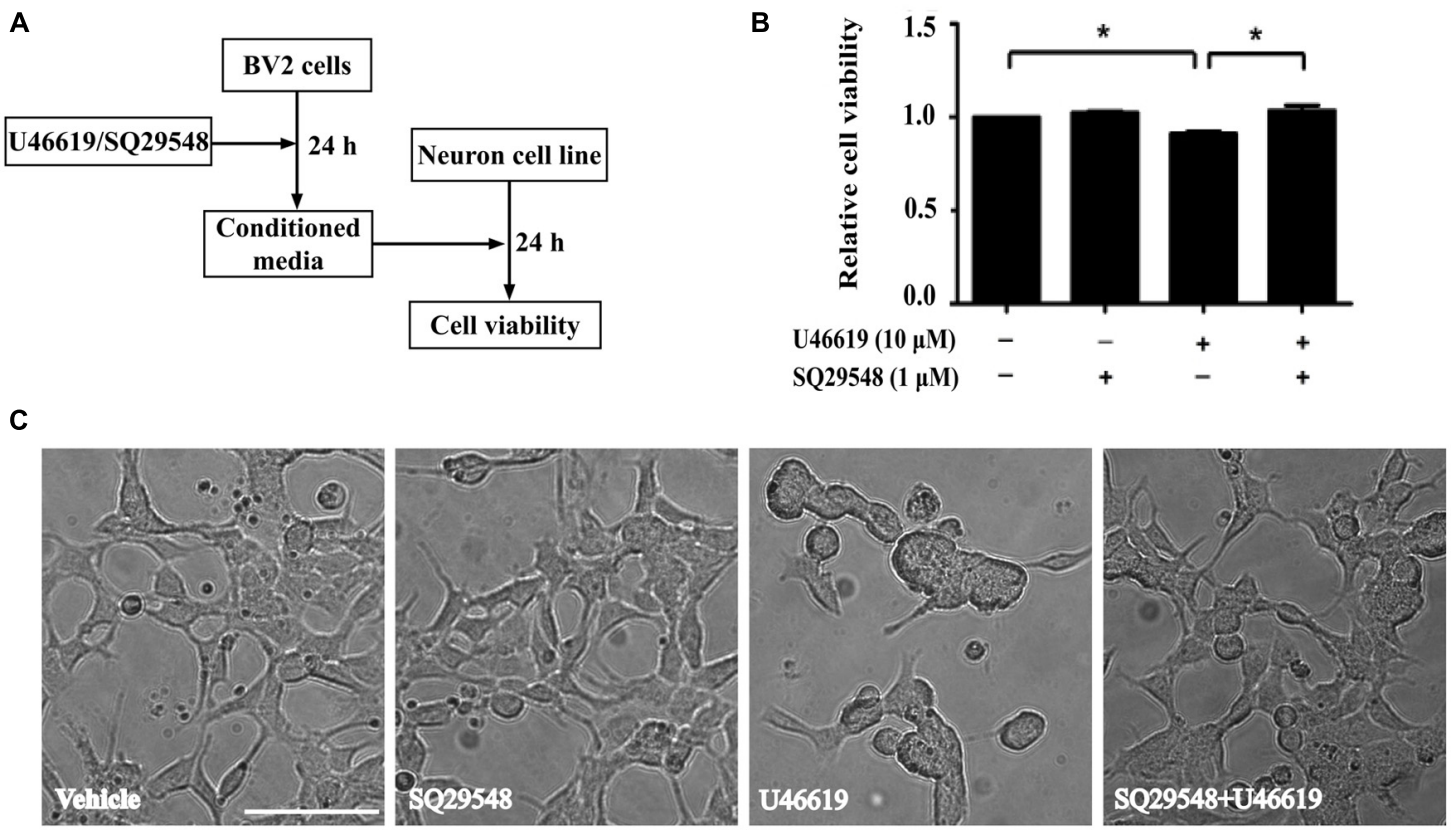
**BV2 conditioned medium by U46619 treatment induces neurotoxicity in SH-SY5Y cells. (A)** The experimental design and procedure. First, BV2 cells were treated by U46619 (10 μM) for 24 h with or without SQ29548 (1 μM, 30 min) pretreatment, and the conditioned medium was collected, which was then applied to SH-SY5Y cells and incubated for 24 h. Effects on SH-SY5Y cells were assessed either by CCK-8 viability assay or microscopy. **(B)** The cell viability of SH-SY5Y cells in various treatment groups was analyzed by CCK-8 assay (Data were normalized against the vehicle-treated group. The data are expressed as the mean ± SEM. ^∗^*P* < 0.05, by one-way ANOVA followed by *post hoc* Turkey’s test). **(C)** Representative microscopic photographs of SH-SY5Y cells in various treatment groups as in **(B)** were shown. Note the differences between the rounded cell shape in the U46619 group and the long stretched cell shape in other three groups. Bar = 100 μm.

The up-regulation of the TP after ischemic stroke and its expression in the microglia prompted us to examine the effects of modulation of TP-mediated signaling. We designed the following experiments to test such effects on neuronal cells (**Figure 3A**). Conditioned medium from BV2 cells treated with TP agonist U46619 should contain secreted proteins and other molecules after activation of TP signaling. A neuronal cell line, human neuroblastoma SH-SY5Y was used to mimic neurons. Such cell line based co-culture experiments are helpful in answering basic questions regarding microglia-neuron interactions (Nam et al., 2013; Zhu et al., 2014). BV2 cells were treated with U46619 (10 μM) for 24 h with or without the TP antagonist SQ29548 (1 μM) pretreatment for 30 min, and the conditioned medium was collected. Then SH-SY5Y cells were incubated with such conditioned medium for 24 h. Finally, the SH-SY5Y cell viability was analyzed. Conditioned medium from U46619-treated BV2 decreased SH-SY5Y cell viability, which was statistically significant compared with vehicle, SQ29548 alone or U46619 plus SQ29548 group (**Figure 3B**). To accompany such findings, cell morphology was directly observed (**Figure 3C**). In all other groups SH-SY5Y cells displayed long processes whereas in the U46619-treated group some neuronal cells were induced to show rounded shapes indicative of cell death (**Figure 3C**). These results suggested that U46619-mediated conditioned medium from BV2 might contain certain factors to reduce SH-SY5Y cell viability. To rule out the possibility that reagents brought with conditioned medium might influence SH-SY5Y cell survival rate, U46619 and SQ29548 were directly added to DMEM medium and placed in incubator for 24 h, and then those mediums were applied to neuronal SH-SY5Y cells. CCK-8 assay showed that there was no statistically significance among groups (**Supplementary Figure S1**). It suggested that reagents brought with conditioned medium did not influence SH-SY5Y cell survival rate.

### U46619 Enhanced Proinflammatory Cytokines and NO Production in BV2 Cells

**FIGURE 4:**
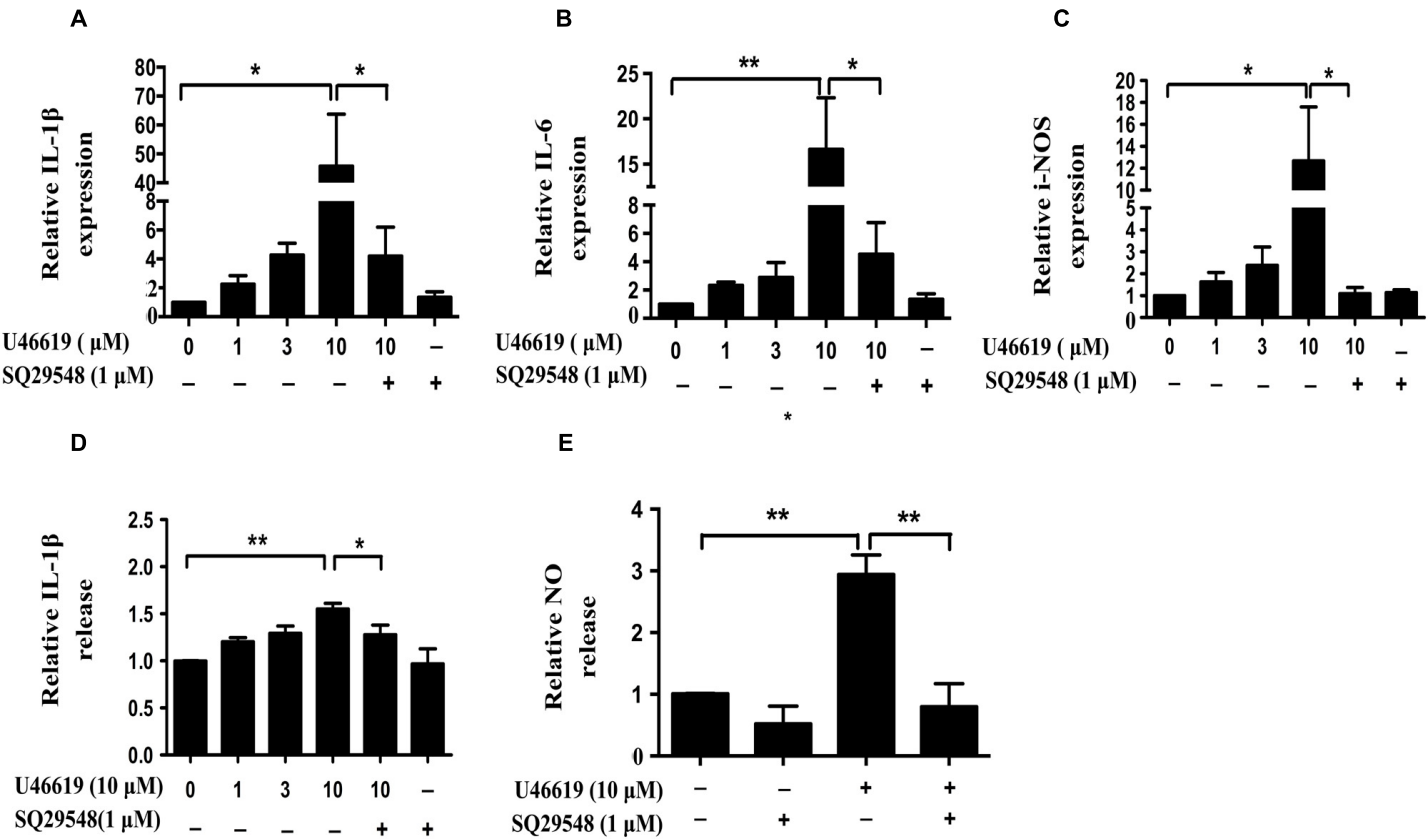
**U46619 enhances proinflammatory cytokines (IL-1β, IL-6) and NO production in BV2 cells. (A)** Relative expression of IL-1β mRNA after U46619 or SQ29548 treatment measured by qRT-PCR. **(B)** Relative expression of IL-6 mRNA under the same conditions. **(C)** Relative expression of iNOS mRNA under the same conditions. **(D)** Relative IL-1β secretion from BV2 cells. After incubation with the indicated concentrations of U46619 for 24 h, IL-1β released from BV2 cells was measured by ELISA. **(E)** Relative NO release from BV2 cells after U46619 or SQ29548 treatment (Data were normalized against the vehicle-treated group. Data were presented as mean ± SEM. ^∗^*P* < 0.05, ^∗∗^*P* < 0.01 by one-way ANOVA followed by *post hoc* Tukey’s test).

Activated microglia release various pro-inflammatory cytokines, chemokines, NO, superoxide (Shao et al., 2013). We then assessed whether TP activation by U46619 could up-regulate cytokine and NO production. Indeed, U46619 largely enhanced transcription of IL1-β, IL-6, and iNOS at mRNA levels (**Figures 4A–C**). Then, we detected IL1-β and NO levels in conditioned medium from BV2 cells. Correspondingly, a significant increase in protein levels of IL1-β had been detected after U46619 stimulation (**Figure 4D**). Pretreatment with TP antagonist SQ29548 (30 min) markedly attenuated IL1-β release (**Figure 4D**). Furthermore, U46619 stimulated an increase of extracellular NO in the conditioned medium, while SQ29548 treatment significantly reduced NO levels (**Figure 4E**). In sum, these results suggested that TP activation could promote proinflammatory factors production and stimulate neuroinflammation, which may explain the reduction of SH-SY5Y cell survival in the above indirect co-culture experiment.

### Extracellular-Signal-Regulated Kinase (ERK) Pathway Mediated the Activation of BV2 Microglia by TP Signaling

**FIGURE 5:**
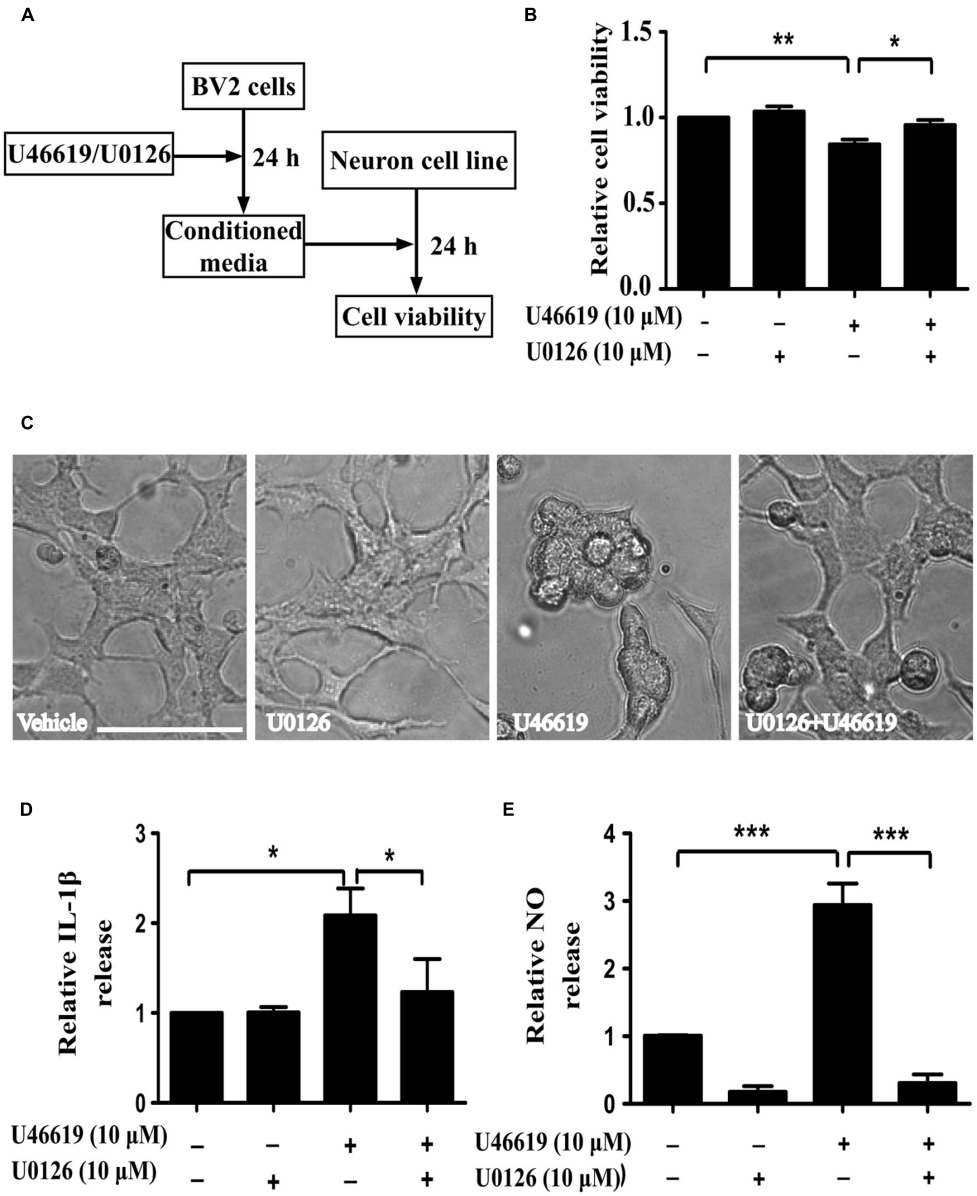
**Extracellular-signal-regulated kinase (ERK) pathway is involved in IL-1β and NO releases by U46619 in BV2 cells. (A)** The experimental design and procedure. First, BV2 cells were treated by U46619 (10 μM) for 24 h with or without U0126 (10 μM, 30 min) pretreatment, and the conditioned media were collected and applied to SH-SY5Y cells. After 24 h incubation, effects on SH-SY5Y cells were assessed either by CCK-8 viability assay or microscopy. **(B)** The viability of SH-SY5Y cells under various treatment conditions was analyzed by CCK-8 assay; and **(C)** cells were also observed by microscopy. **(D)** The effect of U0126 on U46619-induced release of IL-1β in BV2 cells. **(E)** The effect of U0126 on U46619-induced release of NO from BV2 cells. Bar = 100 μm (Data were normalized against the vehicle-treated group. Data were presented as mean ± SEM.^∗^*P* < 0.05, ^∗∗^*P* < 0.01, ^∗∗∗^*P* < 0.001, by one-way ANOVA followed by *post hoc* Tukey’s test).

Activation of the mitogen-activated protein kinases (MAPK) signaling cascade plays an important role in inflammation (Irving and Bamford, 2002). The MAPK family comprises the stress-activated protein kinases/c-Jun N-terminal kinases (SAPK/JNK), the p38 MAPKs, and ERKs. Preliminary experiments had pinpointed the involvement of ERK signaling under TP activation (Honma et al., 2006; Tokue et al., 2009). On this basis, we next investigated whether ERK pathway modulation could affect microglia-neuron interaction after TP activation, following similar experiment design (**Figure 5A**). MEK inhibitor U0126 was used to block ERK activation. BV2 cell were pretreated with U0126 at 10 μM for 30 min, and then stimulated with 10 μM U46619 for 24 h. Conditioned media were collected, applied to SH-SY5Y cells and cultured for 24 h, and then the effects on SH-SY5Y cells were observed. Similar to the result shown in **Figure 3**, U46619-treated conditioned medium from BV2 decreased SH-SY5Y cell viability and induced marked morphological changes, while pretreatment with U0126 partially but significantly attenuated the effect of U46619 (**Figures 5B,C**). DMED mediums directly added with U46619 and U0126 did not influence SH-SY5Y cell survival rate (**Supplementary Figure S1**). Second, we tested whether ERK signaling was involved in IL-1β and NO releases by U46619 in BV2 cells. The results showed that TP activation by U46619 promoted IL-1β protein release in conditioned medium from BV2 cells, while this stimulation was partially but significantly attenuated by U0126 pretreatment (**Figure 5D**). Furthermore, U46619 stimulated an increase of extracellular NO release in the conditioned medium, while U0126 treatment significantly reduced NO levels (**Figure 5E**). Taken together, these results suggested that ERK pathway was involved in IL-1β and NO release induced by U46619 in BV2 cells.

**FIGURE 6:**
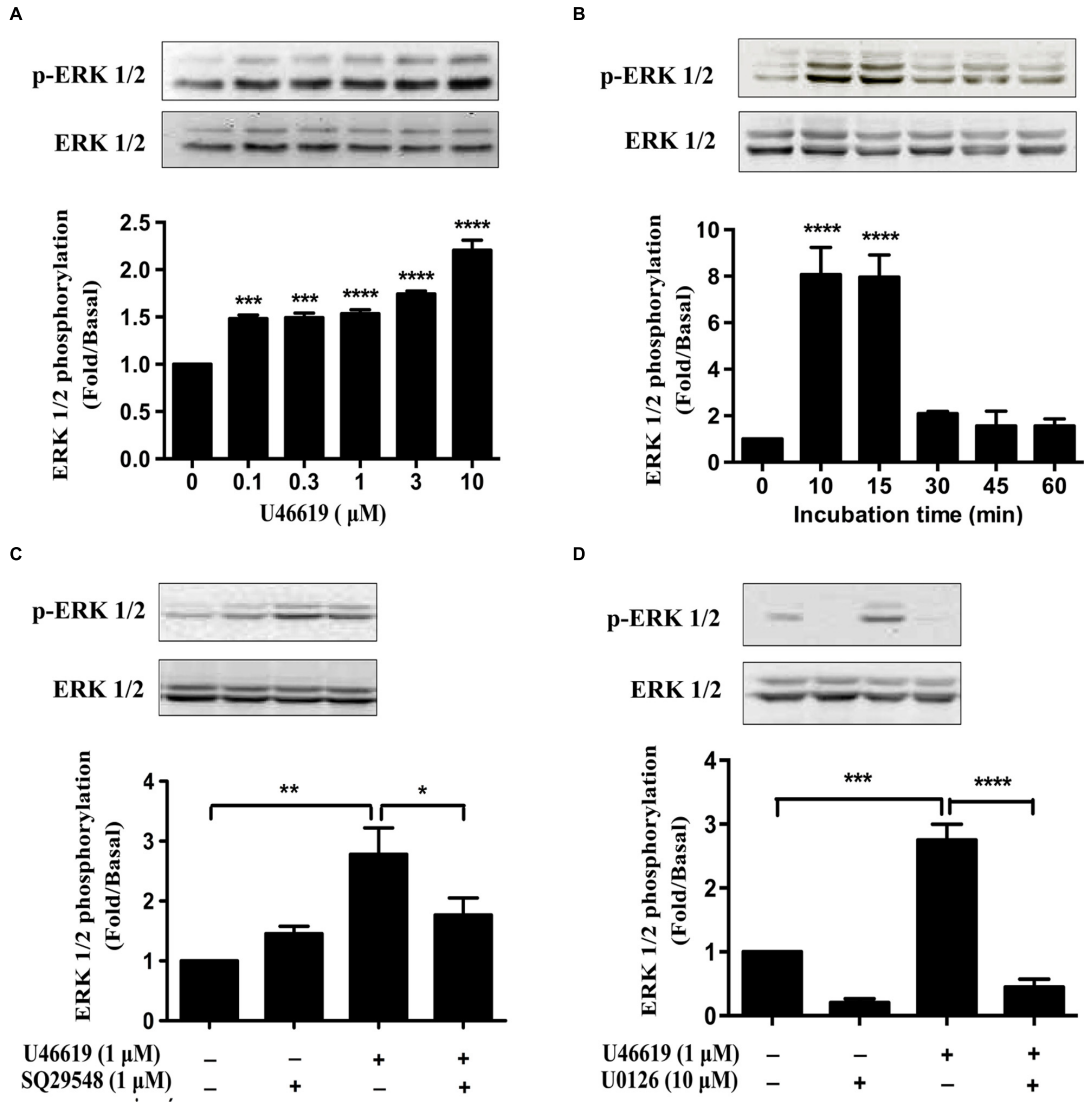
**U46619 stimulates ERK activation in BV2 cells. (A)** Dose-dependent effects of U46619 (0.1–10 μM, 15 min) on ERK activation in BV2 cells. **(B)** Time-dependent effects of U46619 (1 μM, 10–60 min) on ERK activation in BV2 cells. **(C)** The effect of TP inhibitor SQ29548 (1 μM) on U46619-induced ERK activation in BV2 cells. **(D)** The effect of MEK inhibitor U0126 (10 μM) on U46619-induced ERK activation in BV2 cells (The total ERK1/2 and p-ERK1/2 bands were scanned and quantified. The p-ERK1/2 protein levels were normalized to total ERK1/2 protein levels. The basal levels in the vehicle-treated cells were assigned a value of 1.0. The data are expressed as the mean ± SEM. ^∗^*P* < 0.05, ^∗∗^*P* < 0.01, ^∗∗∗^*P* < 0.001, ^∗∗∗∗^*P* < 0.0001 by one-way ANOVA followed by *post hoc* Tukey’s test).

As we have demonstrated that ERK MAPK pathway mediated the IL-1β and NO expression and neuronal toxicity by TP activation, we continued to investigate in detail the dose and temporal profile of ERK phosphorylation stimulated by TP agonist in BV2 cells. Indeed, U46619 caused a dose- and time-dependent stimulation of ERK phosphorylation (**Figures 6A,B**). Elevated ERK phosphorylation occurred after U46619 treatment at a dose as low as 0.1 μM (**Figure 6A**) and maximum activation was achieved within 10–15 min (**Figure 6B**), suggesting a relatively rapid signal response. Consistently, this stimulation was blocked by SQ29548 (1 μM, 30 min) and MEK inhibitor U0126 (10 μM, 30 min) prior to treatment with U46619 (1 μM, 15 min; **Figures 6C,D**). These results demonstrated that TP signaling could induce ERK activation in BV2 cells. As showed in **Figure 5**, ERK pathway was involved in IL-1β and NO release induced by U46619 in BV2 cells. Taken all together, these results supported the notion that ERK pathway mediated the microglial activation by TP agonist U46619.

### ERK Pathway Mediated the Activation of Primary Microglia by TP Signaling

**FIGURE 7:**
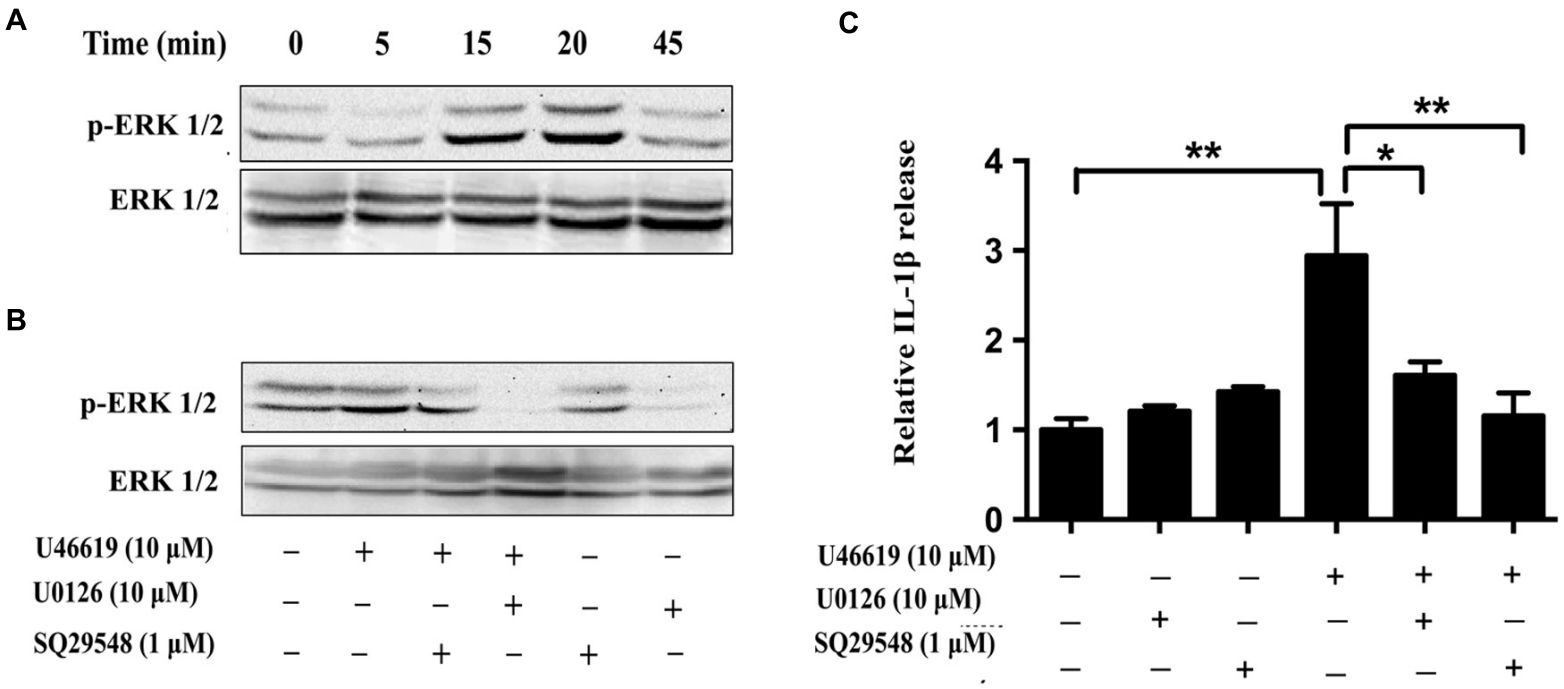
**The effects of TP regulation on ERK activation in primary microglia. (A)** Time-dependent ERK activation by U46619 (1 μM, 5–45 min) in primary microglia. **(B)** Effect of SQ29548 (1 μM, 30 min) and U0126 (10 μM, 30 min) pretreatment on ERK activation by U46619. **(C)** The effect of SQ29548 (1 μM) or U0126 (10 μM) on U46619-induced IL-1β release from primary microglia. After incubation with the indicated concentrations of U46619 for 24 h with or without SQ29548 or U0126 pretreatment, IL-1β released from BV2 cells was measured by ELISA (Data were normalized against the vehicle-treated group. Data were presented as mean ± SEM. ^∗^*P* < 0.05, ^∗∗^*P* < 0.01, by one-way ANOVA followed by *post hoc* Tukey’s test).

We then tried to confirm the results obtained from BV2 cell lines in the primary microglia culture. Consistently U46619 caused a time-dependent stimulation of ERK phosphorylation in primary microglia, with maximum response at 15–20 min after treatment (**Figure 7A**). This stimulation could be blocked by TP antagonist SQ29548 (1 μM, 30 min) and MEK inhibitor U0126 (10 μM, 30 min) prior to treatment with U46619 (**Figure 7B**). Furthermore, TP activation by U46619 promoted IL1-β protein release into conditioned medium from the primary microglia culture, whereas this stimulation was partially attenuated by U0126 pretreatment or completely by SQ29548 (**Figure 7C**). In sum, these results confirmed a physiological role of TP in microglia activation, in which ERK pathway played a significant role.

## Discussion

In the present study, using a well-recognized tMCAO mouse model, we demonstrated that ischemia-reperfusion enhanced TP expression in ipsilateral brain tissue. Immunofluorescence staining revealed that both primary rat microglia and BV2 cells expressed the TP, which was predominantly localized in the plasma membrane. In addition, TP agonist U46619 enhanced IL-1β, IL-6, iNOS mRNA expression and IL-1β, NO release in microglia. The proinflammatory potential of TP was mediated through the ERK phosphorylation. Together these data have suggested that stimulation of TP signaling plays an important role in regulation of microglia activation.

The amount of TXA_2_ is increased dramatically in the central nervous system under pathologic conditions including CI. In fact, activated microglia cells are the principal source of brain-derived TXA_2_ (Fisher, 1993)_._ On the other hand, TP level was up-regulated in brain tissues under pathology conditions such as subarachnoid hemorrhage (Ansar et al., 2010). In our study, we demonstrated for the first time that ischemia-reperfusion increased the TP expression in ipsilateral brain tissue. We also showed that microglia expressed the TP. Thus, after CI, a robust activation of microglia may cause large amount of TXA_2_ secretion, leading to activation of TP on cells around the TXA_2_-producing cells via autocrine or paracrine mechanisms and finally form a vicious circle. Therefore, an investigation of the physiological roles of TP in microglia inflammation will help in providing new targets for the prevention and treatment of CI.

IL-1β contributes to the regulation of various chronic and acute inflammatory responses in central nervous system diseases. Its expression exhibited a biphasic pattern in tMCAO models with a first peak at 1 h after reperfusion and a second peak at 6–24 h (Ceulemans et al., 2010). Activated microglia account for the larger part of IL-1β production in early stage (Utagawa et al., 2008). IL-1β stimulates microglia and astrocytes, leading to an up-regulation of genes encoding for more neurotoxic mediators and inducing a spellbound damaging cycle. Previous studies have demonstrated that overexpression of IL-1β could exacerbate ischemic brain damage, while deficiency in IL-1β has been reported to decrease infarct volume. Caughey et al. (1997) found that TXA_2_ was an important paracrine or autocrine facilitator of TNF-α and IL-1β production in activated human monocytes. Microglia are the resident macrophages in central nervous system. Given those functional TP receptors in activated monocytes, we examined whether TP activation by U46619 could up-regulate IL-1β production in microglia. Consistent with the study in monocytes, we found that U46619 enhanced IL-1β mRNA expression and protein release in microglia, which could be blocked by TP antagonist or ERK inhibition.

NO is an important biomolecule and appears to be mainly toxic in the case of CI (Lakhan et al., 2009). NO can react with superoxide to form peroxynitrite, its reactive metabolite, leading to cellular toxicity. Nitric oxide synthase (NOS) catalyzes the synthesis of NO. However, microglia only express the inducible form of NOS (iNOS; Yenari et al., 2010). After CI, activated microglia enhance the NO production by up-regulating the expression of iNOS mRNA and protein, thus contributing to ischemic brain damage. Inhibition of iNOS with aminoguanidine could reduce infarct volume by about 30% (Iadecola et al., 1995), while iNOS-null mice have shown smaller infarct volume and better neurologic outcomes (Zhao et al., 2000). He et al. (2013) reported that TP activation by I-BOP and U46619 induced an aberrant expression of iNOS in human umbilical vein endothelial cells. Mitsumori et al. (2011) found that TP stimulation in the striatum locally facilitated dopamine overflow evoked by synaptic inputs via NO synthesis in endothelial cells. Lubrano et al. (2008) demonstrated that TP activation enhanced NO production in microvascular endothelium. In the present study, we discovered that U46619 enhanced iNOS mRNA expression and NO release in microglia cells, which could also be attenuated by TP antagonist and ERK inhibitor.

The MAPK family is comprised of the JNK, p38, and ERK (Irving and Bamford, 2002). All three pathways have been described in activated microglia. Various kinds of stimuli, such as lipopolysaccharide (LPS), alpha-synuclein, and amyloid-beta (Aβ), are able to activate MAPK signaling pathways in microglia. In our study, we have focused on the ERK signaling in regulation of microglia. There are a number of literatures in support of such roles of ERK. Kitagawa et al. (1999) revealed that ischemia enhanced ERK expression in brain. Pharmacological inhibition of the ERK signaling pathways improved outcomes in ischemia-reperfusion mouse model (Alessandrini et al., 1999). Previous studies have shown that LPS promoted TNF-α, IL-1β, and IL-6 production in microglia by ERK signaling pathway (Carter et al., 1999; Caivano and Cohen, 2000; Kim et al., 2004). Chan et al. (2001) found that all three pathways (JNK, p38, and ERK) played important regulatory roles in regulation of iNOS and NO expression in mononuclear phagocytes. The ERK pathway may regulate inflammation mainly through its effects on poly (ADP-ribose) polymerase-1 (PARP-1) activation (Kauppinen et al., 2006). PARP-1 is an abundant nuclear enzyme associated with both transcriptional regulation and DNA repair. It can interact with other proinflammatory transcription factors to regulate the inflammatory responses in microglia, including NF-κB, AP-1, etc. (Ha et al., 2002; Yenari et al., 2010). PARP-1 inhibition as well as depletion prevents microglial morphological transformation, secretion of cytokines, migration to lesion site (Yenari et al., 2010).

In our study, we provided evidence to link TP signaling and ERK activation in the microglial cells. This confirms with several previous studies demonstrating that TP can activate ERK signaling pathway in other cell types. Tokue et al. (2009) found that TP promoted phosphoinositide hydrolysis, phosphorylation of ERK, and IL-6 production. Honma et al. (2006) revealed that stimulation of TP causes the glial morphological change with proliferation mainly through ERK signaling pathway. In oligodendrocytes, U46619 was found to increase CREB and ERK phosphorylation, contributing to cell proliferation, survival and gene expression (Lin et al., 2005). Our data showed that TP activation by U46619 promoted IL-1β protein release in microglia, and this stimulation could be significantly attenuated by U0126 pretreatment. From the level of attenuation, ERK pathway was mostly responsible for TP-mediated IL-1β production in microglia. However, it is likely that other signaling pathways could be involved, probably in other cell context. Indeed, Obara et al. (2005) reported that TXA_2_ enhances the IL-6 biosynthesis through the PKA/p38 MAPK/CREB pathway in 1321N1 cells. Zhang et al. (2011) found that TP enhanced protein synthesis in vascular smooth muscle cells via AMP-activated kinase pathway.

There are some limitations in this study. Firstly, although we have demonstrated the involvement of ERK pathway in TP mediated IL-1β and NO production in BV2 cells, the specific signaling pathways downstream of ERK are still poorly understood. The ERK pathway may regulate inflammation through its effects on PARP-1. Whether PARP-1 activation associates with TP mediated microglia inflammation still require further research. Secondly, ERK pathway was only partially responsible for TP mediated IL-1β production in BV2 and primary microglia. The other related signaling pathways need to be evaluated. Thirdly, ischemia-reperfusion enhanced TP expression in ipsilateral brain tissue. However, we only focused on microglia but the role of other types of cells in the brain with regard to elevated TP expression could not be ruled out. We also found that the primary astrocytes express the TP by Western blot (**Supplementary Figure S2**), which warrants further investigation.

## Conclusion

We showed that TP activation increased the expression of proinflammatory cytokines including IL-1β and NO, via ERK signaling pathway in microglia. Our results have suggested a novel physiological role of TP in microglia, which is plausibly a druggable target for the management of neurological diseases like CI.

## Author Contributions

YF, WX, and WY conceived the project, coordinated the study, analyzed the data, and drafted the manuscript. WY, AY, and TZ designed experiments; performed cell line and primary cell culture experiments, generated tMCAO models, carried out analyses involving ELISA, Real-time PCR, immunohistological staining, Western blots; analyzed the data; and drafted the manuscript. JS performed Real-time PCR analysis. TL and XY performed animal experiments. All authors read and approved the final manuscript.

## Conflict of Interest Statement

The authors declare that the research was conducted in the absence of any commercial or financial relationships that could be construed as a potential conflict of interest.

## Supplementary Material

Figure S1 **U46619, SQ29548, and U0126 treatment did not induce cytotoxicity in SH-SY5Y cells.** U46619, SQ29548, U0126, U46619 plus SQ29548 and U46619 plus U0126 were directly added to DMED medium and placed in incubator for 24 h, and then these mediums were applied to neuronal SH-SY5Y cells that had been seeded in 96-well plates. After 24 h, CCK-8 was used to evaluate the changes in SH-SY5Y cell viability.

Figure S2 **Expression of TP in primary astrocytes.** Primary astrocytes were prepared from 24 h neonatal SD rat brains and then cultivated in DMEM/F12 supplemented with 10% fetal calf serum, 1% penicillin/streptomycin. The expression of TP in primary astrocytes was detected by Western blot.
