# Supplementary figures and images for: Thromboxane A2 Receptor Stimulation Enhances Microglial Interleukin-1β and NO Biosynthesis Mediated by the Activation of ERK Pathway
Source: Front Aging Neurosci. 2016 Jan 29;8:8. doi: 10.3389/fnagi.2016.00008 (PMC4731520; doi:10.3389/fnagi.2016.00008)

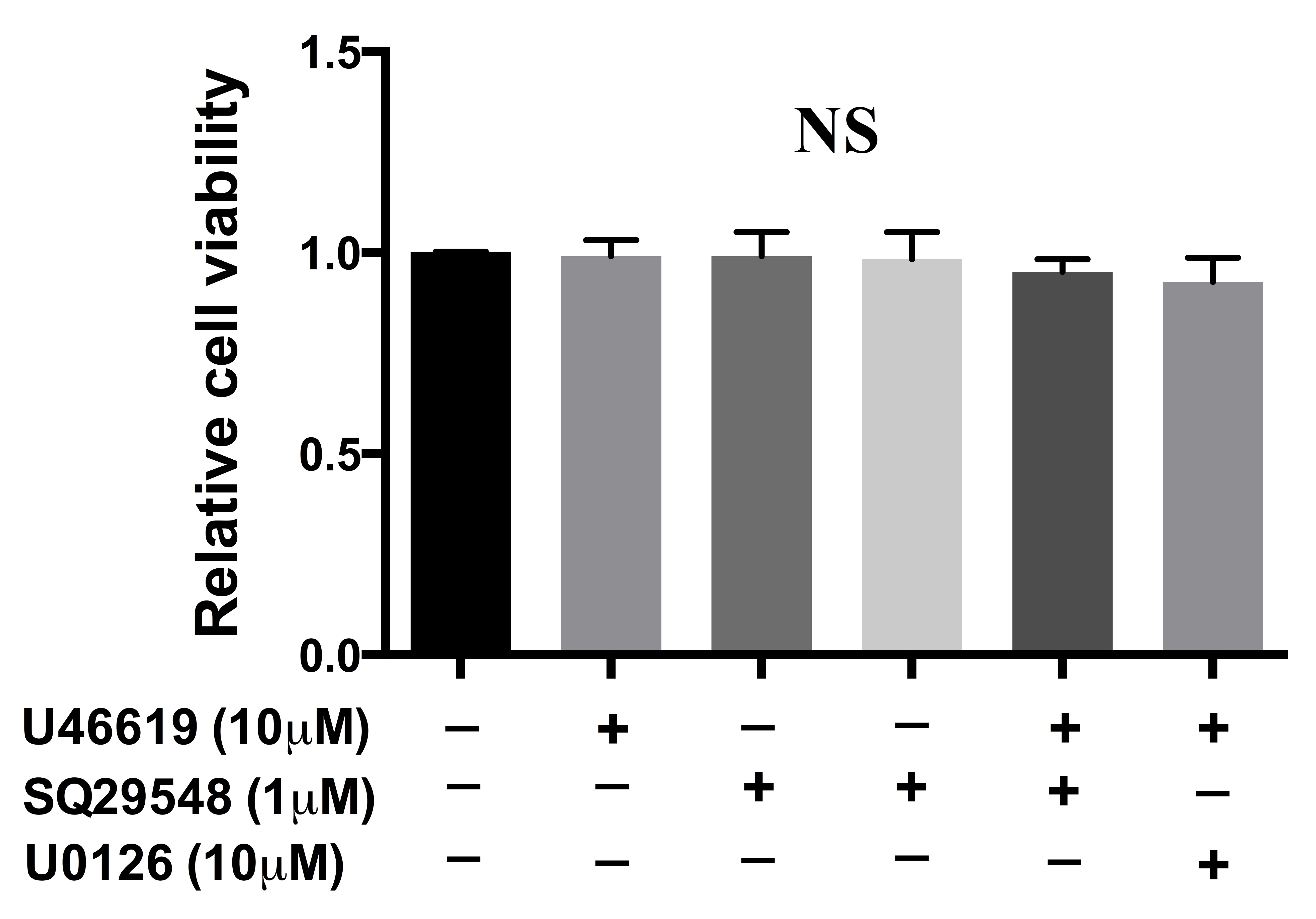

Supplement: Figure S1 — U46619, SQ29548, and U0126 treatment did not induce cytotoxicity in SH-SY5Y cells. U46619, SQ29548, U0126, U46619 plus SQ29548 and U46619 plus U0126 were directly added to DMED medium and placed in incubator for 24 h, and then these mediums were applied to neuronal SH-SY5Y cells that had been seeded in 96-well plates. After 24 h, CCK-8 was used to evaluate the changes in SH-SY5Y cell viability. [file Image_1.JPEG]

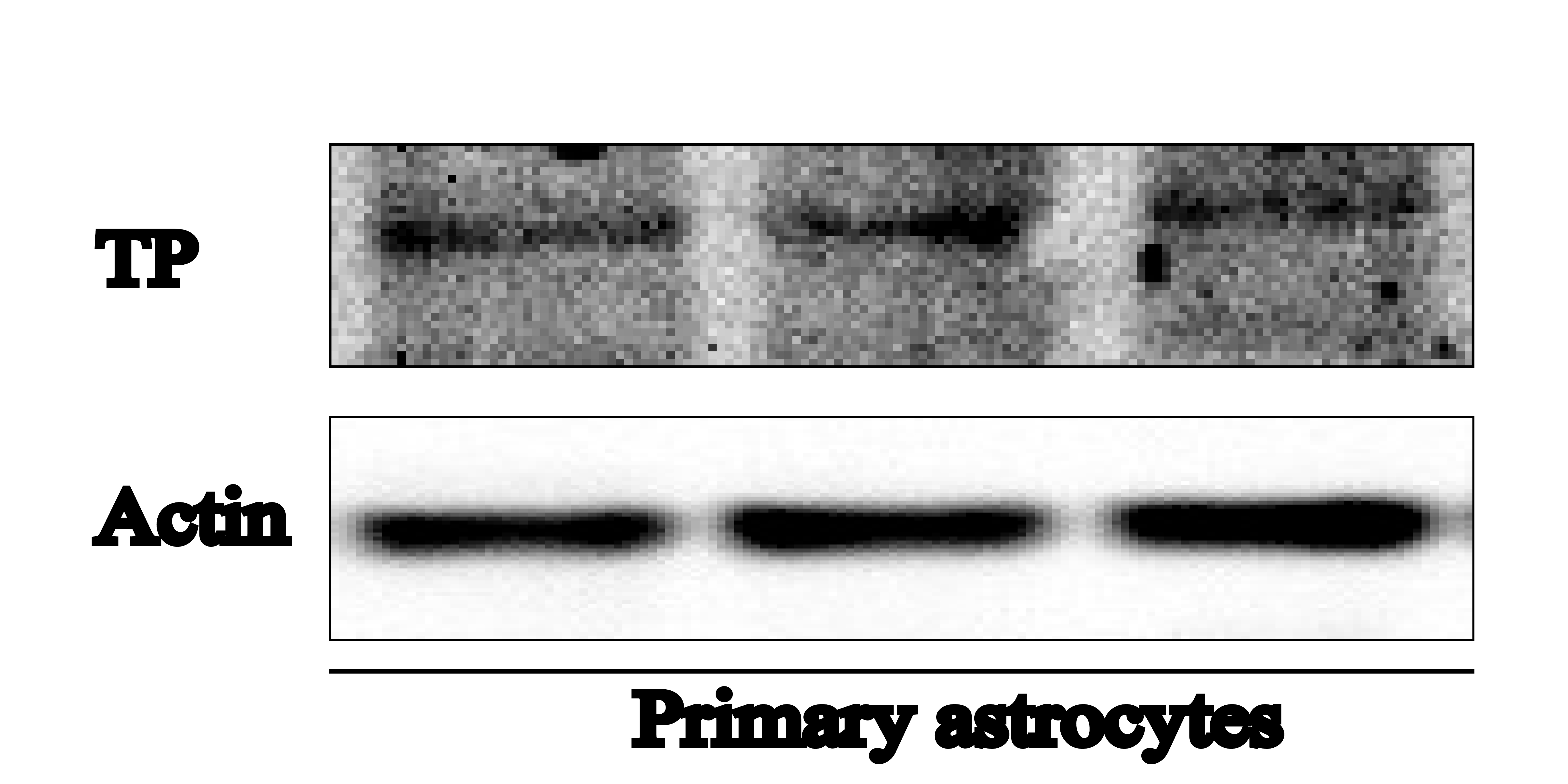

Supplement: Figure S2 — Expression of TP in primary astrocytes. Primary astrocytes were prepared from 24 h neonatal SD rat brains and then cultivated in DMEM/F12 supplemented with 10% fetal calf serum, 1% penicillin/streptomycin. The expression of TP in primary astrocytes was detected by Western blot. [file Image_2.JPEG]
